# Tuberculous Soldiers

**Published:** 1920-02-14

**Authors:** 


THE PUBLIC WELL-BEING—(*continued*)

### Tuberculous Soldiers.

THE members of the Inter-Departmental Committee on Tuberculous Soldiers have addressed a letter to the *Times* in which they show that after a man has been medically treated in the sanatorium and his physical health re-established by curative training in a training colony or section, there is great need of measures to prevent him from drifting back into the anxieties of the wage struggle and possibly unsuitable environment. They propose, therefore, the creation of permanent village settlements.

This threefold conception of sanatorium, training section, and village settlement has had so far its first realisation at Papworth, in Cambridgeshire, but other similar institutions are now in contemplation in Norfolk near Norwich, in East Lancashire, and elsewhere, and the Red Cross are giving substantial help. At Papworth the report states :—

After six months in the training colony section, the patient is offered permanent employment, with housing accommodation, in the village settlement, and in every case up to the present the offer has been accepted. The workshops in which the men have been trained also provide employment for them when they have been transferred to the permanent village settlement. The residents in the village settlement can find employment at the standard rate of wage in the district in carpentry, joinery, including furniture-making, boot-making, tailoring, etc., carried on in suitable workshops and under healthy conditions. In addition, there is dairy work, poultry keeping, and work on the farm, and also in the village shop, which is managed by an ex-patient from the sanatorium.

A large number of the discharged men now patients in the sanatoria, as was shown in evidence before the Committee, welcome the idea of a permanent village settlement, and the authorities at Papworth are flooded with applications at this moment for the cottages in the village settlement.

The report recommends the creation of ten such village settlements, at the cost of, say, one million, to be attached to or built alongside existing sanatoria and to provide accommodation for, say, 2,000 to 3,000 soldiers, with their wives and families.

But so far the Government have given no promise of financial help for these permanent settlements, and the Committee urges that pressure should be brought to bear upon the Government in this respect.

